# Two Decades of Dengue in Indonesia: Long-Term Trends in Incidence, Mortality, and Disability-Adjusted Life Years, 2005–2024

**DOI:** 10.3390/pathogens15040373

**Published:** 2026-03-31

**Authors:** Agus Sudaryanto, Wen-Chun Liao, Yun-Ping Lin, Wen-Chao Ho

**Affiliations:** 1 College of Public Health, China Medical University, Taichung 406040, Taiwan; 2 School of Nursing, Universitas Muhammadiyah Surakarta, Surakarta 57102, Indonesia; 3 School of Nursing, China Medical University, Taichung 406040, Taiwan; 4 Department of Nursing, China Medical University Hospital, Taichung 404332, Taiwan

**Keywords:** dengue, incidence, mortality, case fatality rate, national surveillance, ecological study, disability-adjusted life years, years of life lost, years lived with disability (YLD)

## Abstract

Dengue continues to be a significant public health concern in Indonesia. However, a comprehensive assessment of long-term national trends in disease burden and fatality composition remains to be developed. Ecological time-series analysis for 20 years (2005–2024) was carried out using national surveillance data. Incidence, mortality, and disability-adjusted life year (DALY) rates were calculated using mid-year population estimates. Log-linear regression was applied to estimate the annual percentage change (APC). Joinpoint regression was used to assess potential trend inflection points. Case fatality rate (CFR) trends were examined using negative binomial regression. DALYs were decomposed into years of life lost (YLL) and years lived with disability (YLD). The results indicated that incidence rates did not demonstrate a significant long-term trend, while both mortality and DALY rates declined significantly over time. CFR decreased by approximately 2.2% annually (RR 0.978, 95% CI 0.969–0.987; *p* < 0.001). Although DALYs remained predominantly driven by premature mortality (>98%), the proportional contribution of YLL declined modestly but significantly over time. Despite persistent dengue transmission, fatal severity has substantially decreased in Indonesia over the past two decades (2005–2024). The findings suggest improved clinical outcomes and a modest structural shift in disease burden composition.

## 1. Introduction

Dengue remains one of the most significant mosquito-borne viral diseases globally, with an estimated 390 million infections occurring annually across tropical and subtropical regions [1]. Southeast Asia shares the global burden, and Indonesia is consistently ranked among the countries with the highest dengue incidence worldwide [2,3]. All four types of dengue virus continue to circulate in Indonesia [4,5]. Together with rapid urban growth, changing climate conditions, and high population movement, this has led to repeated dengue outbreaks over the past decades. The epidemiological landscape of dengue has undergone substantial shifts since its initial identification in 1968, characterized by more sporadic outbreaks, evolving serotype prevalence, and an increased average age of patients presenting with dengue hemorrhagic fever [6,7].

In Indonesia, dengue continues to be a major public health problem. Although the government reports the number of cases and deaths each year, a complete understanding of the overall disease burden is still limited. Most studies focus mainly on the number of cases, patterns of spread, or predicting outbreaks [8,9,10,11,12]. Fewer studies examine long-term trends in mortality or the total health loss caused by dengue. Looking only at case numbers may not show the full impact of the disease, especially if changes in case fatality rates and premature deaths are not taken into account.

To better understand the full impact of a disease, researchers often use a measure called Disability-Adjusted Life Years (DALYs). DALYs combine two important parts: Years of Life Lost due to early death (YLL) and Years Lived with Disability or Illness (YLD). By combining these two components, DALYs help compare the overall burden of different diseases and support better decision-making in public health [13,14,15]. Although dengue cases are high in Indonesia, updated national-level DALY estimates based on recent surveillance data are still limited. This gap in comprehensive burden assessment hinders the development of targeted public health strategies and resource allocation for effective dengue control [16]. This study aimed to evaluate long-term trends in dengue incidence, mortality, case fatality rate, and burden of disease of dengue in Indonesia from 2005 to 2024. These findings will support better public health planning and more effective dengue prevention and control strategies in the future [17].

## 2. Materials and Methods

### 2.1. Study Design, Data Sources, and Case Definition

This study employed a national ecological time-series design to examine long-term trends in dengue burden in Indonesia from 2005 to 2024. National surveillance records were used to access annual data on reported dengue cases and deaths. Dengue is a mandated notifiable disease in Indonesia, integrated into a nationwide surveillance system overseen by the Ministry of Health. Since dengue is designated as a notifiable disease in Indonesia, it is formally integrated into the national surveillance system managed by the Communicable Disease Center under the Ministry of Health. The surveillance system has used the World Health Organization (WHO) dengue criteria for symptomatic dengue [18,19]. During two decades (2005–2024), the definitions and criteria of DHF used in this surveillance system have remained constant [6].

Reporting dengue is mandatory in Indonesia within 24 h to 3 days after hospitalization as a requirement of the Ministry of Health of Indonesia by Community Health Centres (Puskesmas) and both private and government hospitals [20]. This mandate requires all healthcare providers in all provinces to evaluate suspected Dengue Hemorrhagic Fever (DHF) cases. Upon initial presentation, healthcare workers must report these cases to district health authorities while waiting for laboratory confirmation. This step triggers epidemiological investigations and vector control interventions, conducted in alignment with national guidelines once serological, virological, or epidemiological links are established.

To refine the diagnosis beyond initial clinical and hematological criteria, cases are categorized based on specific laboratory evidence. A “probable” DHF case is defined by supportive serology, specifically a positive anti-DENV IgM in acute or convalescent samples, or a fourfold increase in IgG titers between these phases. Definitive “confirmed” status is reserved for cases validated through virus isolation or the detection of viral antigens or RNA in the serum. Under current surveillance criteria, only these probable and confirmed cases are ultimately transmitted by district authorities to the central national database for official reporting [6,20]

### 2.2. Burden Estimation

Incidence and mortality rates per 100,000 population were calculated using mid-year population estimates. Disability-adjusted life years (DALYs) were estimated as the sum of years of life lost (YLL) and years lived with disability (YLD) [21,22,23,24,25].

DALY=YLL+YLD

The YLL is the mortality component of the DALY (Disability-Adjusted Life Year). It measures the burden of “premature” death by calculating the years a person would have lived had they not died from a specific cause. The basic formula is

YLL= N× L

• N: Number of deaths due to the specific cause (e.g., Dengue) at a given age.
• L: Standard life expectancy at the age of death (remaining years of life).

Based on the Indonesian life expectancy standard of 72 years (71.9) from the World Bank data [26], a person dying due to dengue at age 15 is estimated to have lost 57 years of healthy life. Using the value of 72 years is a grounded approach that reflects the average life expectancy of Indonesia during the last decade. By using a fixed value of 57 years lost for a 15-year-old, the study avoids “volatility” in the data, ensuring that changes in DALY results are driven by the number of Dengue cases, not by shifting demographic statistics. This study specifically isolates the 15-year-old population as a critical point of analysis. The rationale for this focus is twofold: first, epidemiological trends in Indonesia have shown an ‘age shift,’ where dengue mortality increasingly affects adolescents and young adults rather than being confined to early childhood [20]. While the highest number of reported cases is concentrated among the productive age group (15–44 years) due to high mobility and outdoor exposure, the highest fatality rate is disproportionately found in children under 15 years old. According to the Ministry of Health, children under 15 remain the most vulnerable to death [27]. Second, deaths at age 15 represent a significant loss of potential productive years, which is captured accurately using the YLL.

YLD was estimated using the standard formula:

YLD=I×DW×L

where I represents incident cases of dengue, DW denotes disability weight of dengue, and L indicates disease duration in years. Assumptions: mean duration of symptomatic illness: 10 days (0.027 years) [19], and disability weight: 0.211 [28,29]. The duration of dengue illness typically ranges from 7 to 14 days, including a febrile phase lasting 2–7 days followed by a recovery period, with most patients recovering within one to two weeks [19]. Therefore, a median duration of 10 days was assumed in this study

### 2.3. Trend Analysis

Temporal trends in incidence rate, mortality rate, and DALY rate were assessed using log-linear regression models:

$$\log\left(Rate_{t}\right)=\beta_{0}+\beta_{1}\times Year_{t}$$

The Annual Percent Change (APC) was calculated as

$$APC=\left(e^{\beta_{1}}-1\right)\times 100$$

APC estimates with 95% confidence intervals (CI) were reported.

Joinpoint regression was conducted to identify statistically significant changes in temporal trends. The model allowed detection of inflection points and estimation of segment-specific APCs. The Average Annual Percent Change (AAPC) was calculated to summarize overall trend direction across the 20-year period.

### 2.4. Case Fatality Trend Modeling

To evaluate changes in case fatality over time, Poisson regression was applied with annual deaths as the outcome and the logarithm of reported cases as an offset:

$$\begin{array}{l} Deaths_{t}∼Poisson\left(\lambda_{t}\right)\;\\ \log\left(\lambda_{t}\right)=\alpha +\beta \times Year_{t}+\log\left(Cases_{t}\right) \end{array}$$

The exponentiated coefficient for year was interpreted as the relative change in CFR per year. If overdispersion was detected, a negative binomial model was used.

### 2.5. Sensitivity Analysis

Sensitivity analyses were conducted by varying assumed disease duration (7 days [0.019 years] and 14 days [0.038 years]) to assess the robustness of DALY estimates. The base-case model used an average disease duration of 10 days (0.027 years). A one-way deterministic sensitivity analysis was conducted by varying the assumed duration of illness (7 and 14 days) while holding other parameters constant [30]. DALY estimates under alternative scenarios were compared with the base-case model to evaluate robustness. The relative contribution of fatal and non-fatal components was examined:

$$\%\mathrm{YLL}=\frac{\mathrm{YLL}}{\mathrm{DALY}}\times 100\;\;\%\mathrm{YLD}=\frac{\mathrm{YLD}}{\mathrm{DALY}}\times 100$$

Temporal changes in the proportion of YLL versus YLD were evaluated to explore potential epidemiological transition.

### 2.6. Statistical Software

All analyses were conducted using R software version 4.5.0 (R Foundation for Statistical Computing, Vienna, Austria). Statistical significance was defined as a two-sided *p*-value < 0.05. Detailed code and analysis using R are available in the Supplementary File.

### 2.7. Ethical Considerations

This study utilized aggregated national surveillance data without individual identifiers. Ethical approval was, therefore, not required.

## 3. Results

### 3.1. Descriptive Overall Dengue Burden, 2005–2024

Between 2005 and 2024, a total of 2,477,380 dengue cases and 20,845 deaths were reported nationally. The median annual incidence rate was 45.89 per 100,000 population, with an interquartile range (IQR) of 39.13–60.69, indicating substantial interannual variability in dengue transmission. The highest incidence rate was observed in 2024, reaching 91.93 per 100,000 population. The overall disease burden peaked in 2007, which recorded the highest DALY rate during the study period, as shown in Table 1.

### 3.2. Long-Term Trends Dengue Burden, 2005–2024

Temporal trends in dengue incidence, mortality, and DALY rates were first evaluated using log-linear regression models to estimate annual percent change (APC) over the 2005–2024 period. According to Table 2, the incidence rates did not demonstrate a statistically significant long-term trend (APC = −1.09%; 95% CI: −3.91 to 1.81; *p* = 0.466), indicating substantial interannual fluctuation without consistent directional change. In contrast, mortality rates declined significantly over time (APC = −3.28%; 95% CI: −5.93 to −0.56; *p* = 0.030), corresponding to a sustained reduction in fatal outcomes. DALY rates showed a significant decreasing trend, reflecting the overall disease burden driven primarily by declining mortality.

No clear trend in rate of incidence was identified during the 20-year period (APC −1.09%, 95% CI −3.91 to 1.81; *p* = 0.466). In contrast, the mortality rate significantly exhibited a declining trend (APC −3.28%, 95% CI −5.93 to −0.56; *p* = 0.030). The DALY rate also decreased at an annual percentage change of -3.26% (95% CI approximately −5.9 to −0.5; *p* = 0.031).

**Figure 1:**
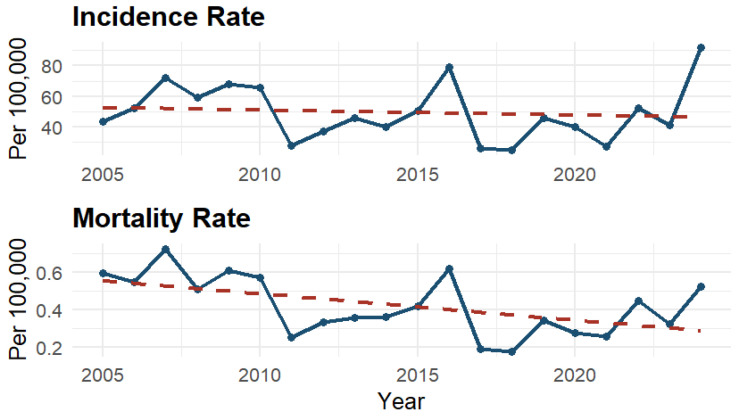
Long-term incidence and mortality rate of dengue in Indonesia over 2005–2024. Blue solid line: rate (incidence and mortality); red dashed line: trend using log-linear regression.

Long-term incidence rates and mortality associated with dengue from 2005 to 2024 are shown in Figure 1. Substantial interannual fluctuations were observed, reflecting the cyclical and outbreak-prone nature of dengue transmission. The absence of a significant long-term decline indicates that transmission intensity remained variable rather than consistently decreasing.

To assess the potential structural changes in transmission dynamics, the incidence rates were transformed with Joinpoint regression. Permutation testing failed to show any statistically significant joinpoints. Given a linear pattern observed for mortality and DALY rates, and no evidence of non-linearity, additional Joinpoint modeling was not pursued for these outcomes.

### 3.3. Case Fatality Rate Trend

Poisson regression analysis demonstrated a highly significant annual decline in case fatality rate (RR 0.976 per year, 95% CI 0.973–0.978; *p* < 0.001), corresponding to an average reduction of 2.4% per year. Model fit for the Poisson regression was assessed using the ratio of residual deviance to degrees of freedom. A value substantially greater than one indicates overdispersion. In this study, the deviance-to-degrees-of-freedom ratio was 16.8, indicating poor fit under the Poisson model. Negative binomial regression was therefore applied.

Based on negative binomial regression, as shown in Table 3, the case fatality rate declined significantly over the study period (RR 0.978 per year, 95% CI 0.969–0.987; *p* < 0.001), corresponding to an average annual reduction of approximately 2.2%. This represents an estimated cumulative decline of roughly 34% over 20 years.

### 3.4. DALY Trend and Decomposition

**Figure 2:**
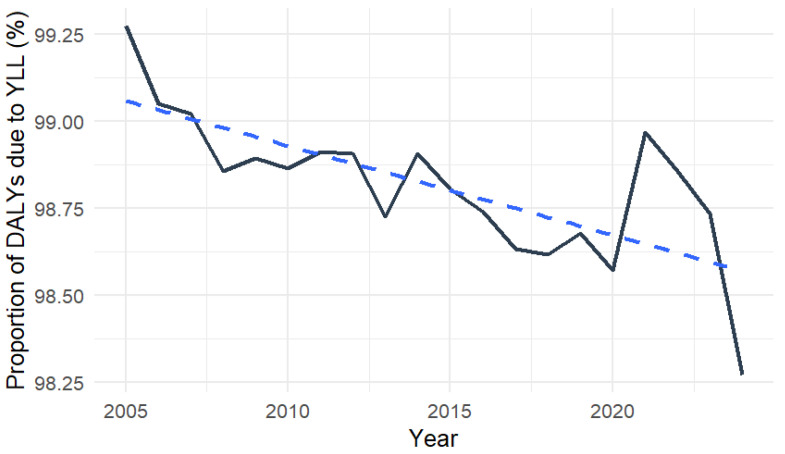
Temporal trend in the proportion of total dengue DALYs attributable to years of life lost (YLL), Indonesia, 2005–2024. The dashed line represents the linear regression trend, and black solid line represents the proportion of YLL in percentage.

To better understand the composition of the dengue burden, DALYs were decomposed into years of life lost (YLL) and years lived with disability (YLD) across the study period. Throughout the study period, YLL accounted for the majority of total DALYs, while YLD contributed only a small fraction of the overall burden. Although annual DALY rates fluctuated substantially, log-linear regression demonstrated a significant overall downward trend. Figure 2 shows the trend of DALY over 20 years.

Linear regression analysis demonstrated a significant decline in the proportional contribution of YLL to total DALYs over time (β = −0.026 percentage points per year; *p* < 0.001). Although YLL remained the dominant component of dengue burden, its relative contribution decreased modestly across the study period.

Sensitivity analysis using alternative disease duration assumptions of 7 and 14 days resulted in minimal changes in DALY estimates. Compared with the base-case scenario, the mean difference in DALY was −0.34% for the 7-day assumption and +0.49% for the 14-day assumption. These results, compared with the base-case model, the 7-day scenario resulted in a mean decrease of 0.34%, whereas the 14-day scenario produced a mean increase of 0.49%. These small differences indicate that the overall burden estimates were robust to reasonable variations in disease duration assumptions.

## 4. Discussion

This 20-year national analysis has identified familiar patterns. Dengue transmission in Indonesia has remained unstable, characterized by recurring fluctuations on an annual incidence basis. Some years showed marked increases followed by sharp declines, illustrating the cyclical pattern of dengue observed in endemic regions [31,32]. The dengue incidence over two decades did not decline consistently. Fluctuating interannual dengue incidence without a trend over time has been reported in endemic countries. Dengue has complex transmission dynamics arising from climate interactions [33,34,35,36,37,38], vector density changes [39,40], population mobility [34,38,41], and viral serotype dominance [34,42,43].

The long-term analysis of the dengue trend not only includes the number and rate of cases, but also how often it proved fatal [44,45]. Between 2005 and 2024, dengue mortality decreased steadily, and the decline in the case fatality rate was even more noticeable. Negative binomial regression showed that the case fatality rate decreased 2.2% each year on average (IRR 0.978; *p* < 0.001). Over 20 years, this gradual yearly decrease resulted in an overall reduction of about one-third. This pattern suggests that improvements happened slowly and consistently over time, rather than because of one major event. The patient case fatality rates have improved through a series of continuous advancements in clinical protocols, more efficient early detection, and enhanced healthcare responses [46].

This difference between the number of infections and the severity of outcomes is very important. Because incidence did not decrease significantly, the reduction in mortality was likely not caused by fewer people being exposed to dengue. Instead, the findings suggest improvements in medical care, earlier detection of dengue cases, better referral systems, and an improvement in the overall health system response. Even if dengue transmission continues, better or decreased case fatality rates can reduce the overall burden of the disease at the population level.

The DALY analysis is discussed in detail. Throughout the study period, premature mortality accounted for the majority of total DALY. In practical terms, dengue burden in Indonesia has been driven far more by deaths than by disability. It is similar to earlier work from Brazil that found that most dengue DALYs derived from fatal and severe cases, highlighting that years of life lost were the principal driver of burden [47]. Although the proportional contribution of YLL declined gradually over time, mortality remains the dominant component of burden [48]. The small change observed likely reflects improvements in the case fatality rate rather than major changes in non-fatal disease impact.

No significant joinpoints were detected in the incidence rate, indicating that changes occurred gradually over time. There was no clear change in the route of transmission. Its mean, dengue has continued to follow its usual endemic pattern, with cases increasing and decreasing in cycles [49]. This highlights the difficulty of controlling mosquito populations in tropical environments, such as in Indonesia [50].

This study has several limitations that should be considered. First, the analysis was based on aggregated national surveillance data and did not include age-specific mortality information. Because of this, years of life lost (YLL) were estimated using a simplified life expectancy method. This approach does not fully reflect differences in remaining life expectancy across age groups. However, the main goal of this study was to examine long-term trends over time rather than to produce precise burden estimates comparable to full Global Burden of Disease analyses [22]. The study did not analyze differences in DALY between children and adults, nor did it distinguish between severe and mild dengue cases [51]. The estimates represent the overall national burden and do not capture variations in disease severity or age-related risk. Future studies using more detailed, age-stratified, and clinical severity data would provide a clearer understanding of how dengue burden differs across population groups.

Sensitivity analyses showed that changing reasonable assumptions about disease duration had only a very small effect on the DALY results. This indicates that the overall findings are stable and reliable. Finally, because this was an ecological study using population-level data, it cannot determine cause-and-effect relationships at the individual level.

These limitations should be taken into account; however, this study possesses important strengths. It draws on data collected over two decades, which allows us to see how dengue patterns have developed over time rather than only looking at shorter trends. The study also used different types of analysis to view the data from multiple perspectives. These included examining overall trends, looking at possible changes in direction over time, analyzing case fatality rates, and disaggregating. Using these methods in combination, the study shows a clear and comprehensive picture of how dengue has evolved at the national level.

## 5. Conclusions

Over the past 20 years, dengue transmission in Indonesia has fluctuated, with case numbers rising and falling from year to year and no clear long-term decrease in incidence. In contrast, both mortality rates and DALY rates showed steady and significant declines during the same period. The gradual reduction in the case fatality rate suggests that patient care and the health system’s response have improved over time rather than through sudden changes. Although premature deaths still accounted for most of the total DALYs, their proportion decreased slightly over time. This indicates gradual improvements in survival and possible changes in disease severity. Sensitivity analyses confirmed the robustness of DALY estimates under alternate assumptions about duration. Overall, these data argue that although dengue transmission is persistent, its fatal impact has decreased over time. Continued investment in vector control, early detection and resilience of clinical care systems will be needed to transform gains in survival into wider reductions in overall disease burden.

## Figures and Tables

**Table 1:** Descriptive Epidemiological Indicator (2005–2024).

Indicator	Value	Year
Total reported cases	2,477,380	2005–2024
Total deaths	20,845	2005–2024
Median incidence rate (per 100,000)	45.89	2005–2024
Interquartile range (IQR) incidence	39.13–60.69	2005–2024
Highest incidence year	91.93	2024
Highest DALY rate year	41.78	2007

**Table 2:** Long-Term Trends in Dengue Epidemiological Indicators, 2005–2024.

Outcome	APC (95% CI)	*p*-Value ^1^	Description
Incidence rate	−1.09 (−3.91 to 1.81)	0.466	not significant
Mortality rate	−3.28 (−5.93 to −0.56)	0.030	significant
DALY rate	−3.26 (−5.90 to −0.54)	0.031	significant

^1^ Note: Evaluated using log-linear regression models.

**Table 3:** The Case Fatality Rate Trend of Dengue in Indonesia during 2005–2024.

Variable	RR	95% CI	*p*-Value
Year (per 1-year increase)	0.978	0.969–0.987	<0.001

Note: Evaluated using negative binomial regression models.

## Data Availability

The data is publicly available in the Figshare repository; https://doi.org/10.6084/m9.figshare.31441663 (accessed on 20 February 2026). Further inquiries can be directed to the corresponding authors.

## Supplementary Materials

The following supporting information can be downloaded at: https://www.mdpi.com/article/10.3390/pathogens15040373/s1, File S1. R Scripts.

## Abbreviations

The following abbreviations are used in this manuscript:

DALY	Disability-Adjusted Life Years
YLL	Years of Life Lost
YLD	Years Lived with Disability
IRR	Incidence Rate Ratio
APC	Annual Percentage Change
CFR	Case Fatality Rate
IQR	Inter Quartile Range
DHF	Dengue Hemorrhagic Fever
