# Supplementary material for: Two Decades of Dengue in Indonesia: Long-Term Trends in Incidence, Mortality, and Disability-Adjusted Life Years, 2005–2024
Source: Pathogens. 2026 Mar 31;15(4):373. doi: 10.3390/pathogens15040373 (PMC13119181; doi:10.3390/pathogens15040373)
Supplement: Supplementary file 1 [file pathogens-15-00373-s001.zip › pathogens-4204716-supplementary.pdf]

Supplementary File:

1. #R Preparation

```
install.packages("readxl")
install.packages("ggplot2")
install.packages("MASS")

# Load
library(readxl)
library(ggplot2)
library(MASS)
```

2. LOAD DATA

```
setwd("D:/dengue_trend")
data <- read_excel("Cases.xlsx")
```

3. LOG-LINEAR REGRESSION

```
#dengue incidence

model_inc <- lm(log(Incidence) ~ Year, data = data)


beta_inc <- coef(model_inc)[2]
se_inc <- summary(model_inc)$coefficients[2,2]
p_inc <- summary(model_inc)$coefficients[2,4]


APC_inc <- (exp(beta_inc) - 1) * 100
lower_inc <- (exp(beta_inc - 1.96 * se_inc) - 1) * 100
upper_inc <- (exp(beta_inc + 1.96 * se_inc) - 1) * 100

APC_inc
lower_inc
upper_inc
p_inc

#dengue mortality

model_mort <- lm(log(Mortality) ~ Year, data = data)

beta_mort <- coef(model_mort)[2]
se_mort <- summary(model_mort)$coefficients[2,2]
p_mort <- summary(model_mort)$coefficients[2,4]
```

```
APC_mort <- (exp(beta_mort) - 1) * 100
lower_mort <- (exp(beta_mort - 1.96 * se_mort) - 1) * 100
upper_mort <- (exp(beta_mort + 1.96 * se_mort) - 1) * 100

APC_inc
lower_inc
upper_inc
p_inc

#dengue DALY

model_daly <- lm(log(DALY) ~ Year, data = data)

beta_daly <- coef(model_daly)[2]
se_daly <- summary(model_daly)$coefficients[2,2]
p_daly <- summary(model_daly)$coefficients[2,4]

APC_daly <- (exp(beta_daly) - 1) * 100
lower_daly <- (exp(beta_daly - 1.96 * se_daly) - 1) * 100
upper_daly <- (exp(beta_daly + 1.96 * se_daly) - 1) * 100

APC_daly
lower_daly
upper_daly
p_daly
```

4. CFR + NEGATIVE BINOMIAL

```
model_nb <- glm.nb(Deaths ~ Year + offset(log(Cases)), data = data)
summary(model_nb)
```

5. CFR

```
model_cfr <- glm(Deaths ~ Year + offset(log(Cases)),
         family = poisson(link = "log"),
         data = data)
summary(model_cfr)
beta_cfr <- coef(model_cfr)[2]
se_cfr <- summary(model_cfr)$coefficients[2,2]
p_cfr <- summary(model_cfr)$coefficients[2,4]

RR <- exp(beta_cfr)
lower_rr <- exp(beta_cfr - 1.96 * se_cfr)
upper_rr <- exp(beta_cfr + 1.96 * se_cfr)
```

```
RR
lower_rr
upper_rr
p_cfr
```

6. Model CFR

```
model_cfr <- glm(Deaths ~ Year + offset(log(Cases)),
          family = poisson(link = "log"),
          data = data_cfr)

summary(model_cfr)
beta_cfr <- coef(model_cfr)[2]
se_cfr <- summary(model_cfr)$coefficients[2,2]
p_cfr <- summary(model_cfr)$coefficients[2,4]

RR <- exp(beta_cfr)
lower_rr <- exp(beta_cfr - 1.96 * se_cfr)
upper_rr <- exp(beta_cfr + 1.96 * se_cfr)

RR
lower_rr
upper_rr
p_cfr

# confidence interval
beta_cfr <- -0.024208
se_cfr <- 0.001154

RR <- exp(beta_cfr)
lower_rr <- exp(beta_cfr - 1.96 * se_cfr)
upper_rr <- exp(beta_cfr + 1.96 * se_cfr)

RR
lower_rr
upper_rr
```

7. Overdispersion test

```
deviance(model_cfr) / df.residual(model_cfr)
1 → (Poisson valid)
```

1–1.5 → acceptable
2 → overdispersion
→ Use Negative Binomial

8. Negative binomial

```
model_nb <- glm.nb(Deaths ~ Year + offset(log(Cases)),
              data = data_cfr)
summary(model_nb)

beta_nb <- coef(model_nb)[2]
se_nb <- summary(model_nb)$coefficients[2,2]
p_nb <- summary(model_nb)$coefficients[2,4]

RR_nb <- exp(beta_nb)
lower_nb <- exp(beta_nb - 1.96 * se_nb)
upper_nb <- exp(beta_nb + 1.96 * se_nb)

RR_nb
lower_nb
upper_nb
p_nb
```

9. DALY Decomposition

```
data$prop_YLL <- (data$YLL / data$DALY) * 100
data$prop_YLD <- (data$YLD / data$DALY) * 100

head(data[, c("Year","prop_YLL","prop_YLD")])

data[data$Year %in% c(2005, 2024),
   c("Year","prop_YLL","prop_YLD")]

model_yll_prop <- lm(prop_YLL ~ Year, data=data)
summary(model_yll_prop)

#graph
plot(data$Year, data$prop_YLL, type="l", lwd=2,
   ylab="Percentage of DALY",
   xlab="Year")

lines(data$Year, data$prop_YLD, col="red", lwd=2)
```

```
legend("topright",
      legend=c("YLL (%)","YLD (%)"),
      col=c("black","red"),
      lty=1, lwd=2)
```

10. #DALY Decomposition, YLL Proportion

```
data$prop_YLL <- (data$YLL / data$DALY) * 100

model_prop <- lm(prop_YLL ~ Year, data = data)
summary(model_prop)
#Figure DALY Decomposition
# the proportion ready
data$prop_YLL <- (data$YLL / data$DALY) * 100
data$prop_YLD <- (data$YLD / data$DALY) * 100

library(ggplot2)
library(tidyr)

# long format
data_long <- data %>%
  select(Year, prop_YLL, prop_YLD) %>%
  pivot_longer(cols = c(prop_YLL, prop_YLD),
               names_to = "Component",
               values_to = "Percentage")

# Rename label
data_long$Component <- factor(data_long$Component,
                              levels = c("prop_YLL","prop_YLD"),
                              labels = c("YLL (%)","YLD (%)"))

# Plot
library(dplyr)
library(tidyr)
library(ggplot2)

ggplot(data, aes(x = Year, y = prop_YLL)) +
  geom_line(size = 1.2, color = "#2C3E50") +
  geom_smooth(method = "lm", se = FALSE, linetype = "dashed") +
  labs(x = "Year",
      y = "Proportion of Total DALYs Attributable to YLL (%)",
```

```
    title = "Temporal Change in the Proportion of DALYs Due to Premature Mortality")
+
  theme_minimal(base_size = 14)
```

11. #Figure → Incidence & Mortality

```
par(mfrow = c(2,1), mar = c(4,4,2,1))

plot(data$Year, data$Incidence,
    type = "b", pch = 16,
    xlab = "Year",
    ylab = "Incidence rate per 100,000")

abline(lm(Incidence ~ Year, data = data), lty = 2)

plot(data$Year, data$Mortality,
    type = "b", pch = 16,
    xlab = "Year",
    ylab = "Mortality rate per 100,000")

abline(lm(Mortality ~ Year, data = data), lty = 2)
```

12. #Sensitivity Analysis

```
L_base <- 10/365
L_7 <- 7/365
L_14 <- 14/365

#Disability weight (dengue):
# Parameter
DW <- 0.211
L_base <- 10/365
L_7 <- 7/365
L_14 <- 14/365

# YLD alternatif
data_cfr$YLD_7 <- data_cfr$Cases * DW * L_7
data_cfr$YLD_14 <- data_cfr$Cases * DW * L_14

# DALY alternatif
data_cfr$DALY_7 <- data_cfr$YLL + data_cfr$YLD_7
data_cfr$DALY_14 <- data_cfr$YLL + data_cfr$YLD_14
```

```
# H % DALY base-case
data_cfr$diff_7 <- ((data_cfr$DALY_7 - data_cfr$DALY) / data_cfr$DALY) * 100
data_cfr$diff_14 <- ((data_cfr$DALY_14 - data_cfr$DALY) / data_cfr$DALY) * 100

# mean_diff
mean_diff_7 <- mean(data_cfr$diff_7)
mean_diff_14 <- mean(data_cfr$diff_14)

mean_diff_7
mean_diff_14
```
